# Prospective comparison of slow-pull and standard suction techniques of endoscopic ultrasound-guided fine needle aspiration in the diagnosis of solid pancreatic cancer

**DOI:** 10.1186/s12876-018-0921-9

**Published:** 2019-01-09

**Authors:** Renáta Bor, Béla Vasas, Anna Fábián, Anita Bálint, Klaudia Farkas, Ágnes Milassin, László Czakó, Mariann Rutka, Tamás Molnár, Mónika Szűcs, László Tiszlavicz, László Kaizer, Sándor Hamar, Zoltán Szepes

**Affiliations:** 1 First Department of Medicine, University of Szeged, Korányi Fasor 8-10, Szeged, 6720 Hungary; 2 Department of Pathology, University of Szeged, Szeged, Hungary; 3 Department of Medical Physics and Informatics, University of Szeged, Szeged, Hungary

**Keywords:** Pancreatic cancer, Sampling, Endoscopic ultrasound, Endosonography, EUS-FNA

## Abstract

**Background:**

The usage of endoscopic ultrasound-guided fine needle aspiration (EUS-FNA) for the diagnosis of solid pancreatic cancer is increasing, however mainly retrospective studies are available about the detailed methods of sampling.

**Methods:**

To compare prospectively the diagnostic yield of EUS-FNA samples obtained with slow-pull (SP) and with standard suction technique (SS).

**Results:**

EUS-FNA sampling was diagnostic in 72 of 92 cases (78.3%). Diagnostic yield was 67.4% in the SS and 65.2% in the SP group. The number of smear pairs (1.84 vs. 3.56; *p* < 0.001) and blood contamination (1.50 vs. 2.19; *p* < 0.001) were significantly higher in the SS group, which resulted in lower rate of diagnostic samples (41.8% vs. 30.0%; *p* = 0.003). There was no difference in the cellularity (1.58 vs. 1.37; *p* = 0.2554), or in the sensitivity and specificity in the identification of malignancy between SP and SS subgroups (69.9, 100% vs. 73.5, 100%). Histological samples were obtained in 60 cases (with SP: 49 cases; with SS: 46 cases). There was no difference in the diagnostic yield of histological samples between the groups (63 and 58.7%).

**Conclusion:**

The diagnostic yield, the cellularity of smears and the rate of acquiring sufficient histological material are similar in the SP and SS group, but due to lower bloodiness and decreased number of slides, the pathological diagnosis is faster and more cost-effective.

## BACKBROUND

Endoscopic ultrasound-guided fine needle aspiration (EUS-FNA) is an accurate and safe diagnostic modality, which by now has become the first line sampling procedure for the histological/cytological diagnosis of solid pancreatic cancer [1, 2]. The diagnostic yield of EUS-FNA in differentiating benign and malignant pancreatic lesions varies widely, and it is substantially influenced by the experience of the endosonographer and the pathologist, the tumor characteristics, sampling technique, processing of smears [3]. The pooled sensitivity and specificity of malignant cytology is 83–95% and 71–100%, respectively [4, 5].

Despite the increasing use of EUS-FNA, there are no evidence-based recommendations about the detailed technique and processing of smears, therefore, they vary substantially across medical centers. The optimal sampling technique is expected to produce samples of satisfactory quality with high cellularity and low blood contamination [6]. The high number of smears is one of major limitations of EUS-FNA, because it increases costs and the length of pathological evaluation. These quality features are influenced by the vascularity and stiffness of the tumor, as well as by the needle and suction characteristics. The suction force for sampling can be generated by multiple different ways. The slow pull out of stylet during the sampling generates a small suction/capillary force (stylet slow-pull technique; SP). In contrast, greater suction/vacuum is created attaching a 5 or 10 mL syringe to the hub of the needle after quick removal of the stylet (standard suction; SS). In addition, many subtypes of the two techniques are known based on the duration (constant, intermittent) and/or the strength of suction force, and how it has been created (with vacuum syringe or manually). The technical guideline of European Society of Gastrointestinal Endoscopy (ESGE) recommends continuous suction for EUS-FNA of pancreatic solid masses and cystic lesions [7, 8]. Recently published comparison studies are questioning this, although their results are contradictory. Some studies suggest that the quality of smears obtained by SP is better compared with SS, but others disprove this [9–11].

Therefore, the aim of our prospective study was to compare the diagnostic yield of EUS-FNA samples obtained by SS and SP in patients with suspected malignant pancreatic lesions based on the number of diagnostic smear pairs, bloodiness and cellularity.

## Methods

This prospective comparison study was carried out between January 2014 and June 2016 in a tertiary level referral medical center with collaboration between the pathology and gastroenterology department. 92 patients were enrolled who underwent EUS-FNA sampling due to suspected pancreatic cancer. The inclusion criteria were the following: 1) previously identified solid pancreatic lesions by cross-section imaging modalities which were suspicious for malignancy; 2) unresectable disease due to local invasion, dissemination to distant organs, severe comorbidity or poor general condition of the patient, or cases when the patient did not consent to surgery; 3) the cytological or histological verification of pancreatic cancer was necessary for the selection of adequate further therapy.

The study was approved by the Regional and Institutional Human Medical Biological Research Ethics Committee of the University of Szeged (ethics approval number: 3679 SZTE). The study is carried out under the declaration of Helsinki.

### EUS-FNA sampling, preparation of specimens

EUS-FNA examinations were performed by the same two investigators under intravenous premedication with 5–15 mg midazolam, 20 mg butylscopolamine and 10–20 mg nalbuphine. Linear echoendoscope (Olympus GF-UCT 140; Olympus GF-UCT 160; Olympus Optical, Tokyo, Japan) and 22G FNA needles (Echotip Ultra; Cook Ireland Ltd., Limerick, Ireland; EZ Shot 2, Olympus Optical, Tokyo, Japan) were applied for the sampling. After the determination of the optimal puncture site, the needle was inserted under continuous real-time ultrasound guidance to the target lesion and approximately 10 to 15 back-and-forth movements were done in a fanning manner. 2–2 punctures using SP and SS technique were proposed to be carried out with the same needle from the same lesion, but reduction or increase of the punctures’ number was allowed if the examiner considered it necessary based on the quality of the obtained specimen. In case of SP, suction force is generated by the slow pull out of stylet during the back-and-forth movements of needle. In case of SS technique, 5 ml syringe was attached to the hub of the needle after quick removal of the stylet to create greater suction force compared to SP. The puncture was considered technically successful if it yielded any grossly or microscopically identifiable cells or tissue fragments regardless of their diagnostic value. It means that the suction force was strong enough for mobilizing cellular material from the target organ. The aspirated specimen was expressed onto glass slides by reinserting the stylet into the needle. The prepared smears were fixed in 96% methanol at least for 10 min. The grossly visible, whitish, yellowish or reddish pieces of tissue were placed in 10% buffered formalin. After the repeated removal of the stylet, the residual aspirated material was flushed out from the needle with saline and 5 ml air to a native sampling tube. This procedure flushed out the residual specimen from the needle. Rapid on-site evaluation (ROSE) was unavailable.

After EUS-FNA sampling, patients were closely observed for 24 h, and blood samples were taken for detection of procedure related complications (elevated amylase level, acute pancreatitis, infection, bleeding, etc.).

### Pathological assessment

All cytological smears were stained by hematoxylin-eosin (HE); immunocytochemistry was performed only in selected cases on smears with high cellularity. The fluid in the native sampling tube obtained by flushing the EUS-FNA needle with saline was centrifuged, and smears or paraffin-embedded cell block samples were prepared. The quality of smears was assessed based on the semiquantitative scale of bloodiness and cellularity *(*Table 1*)*, furthermore the number of diagnostic slide pairs was determined. The formalin-fixed and paraffin-embedded (FFPE) tissues were processed using the standard protocol for endoscopic biopsies: standard staining with HE supplemented by mucin stain (periodic acid-Schiff reaction after Alcian blue staining, pH 2.5, PAB) and immunohistochemical testing (CK7, CK20, MUC5AC, CDX-2, chromogranin A, synaptophysin, etc.). The efficiency and diagnostic value of EUS-FNA sampling was determined based on the classification of Papanicolaou Society [12]. *(*Table 2*)* The sampling was considered diagnostic if it clearly confirmed the presence of non-neoplastic (Papanicolaou II. category) or neoplastic pancreatic lesion (Papanicolaou IV., and VI. categories), or when the cytopathologist had a high degree of certainty of the presence of carcinoma in clinically unequivocally malignant-appearing tumors (Papanicolaou V. category).

**Table 1:** Bloodiness of smears

Cellularity of smears	
0 - Acellular	No or only a few tumor cells
1 – Low	< 2 clusters of malignant cells with a minimum 10 tumor cells
2 – Medium	2-4 clusters of malignant cells with a minimum 10 tumor cells
3 – High	> 4 clusters of malignant cells with a minimum 10 tumor cells
Bloodness of smears	
0 - Absence	No or minimal blood contamination
1 – Mild	A few blood cells which do not interfere with pathological evaluation
2 – Moderate	Partially covered by blood cells, but pathological evaluation is possible
3 – Severe	Covered by blood cells which interfere with pathological evaluation

**Table 2:** Classification of Papanicolaou Society for assessment of cytological sampling of the pancreatobiliary system. (NET - neuroendocrine tumor; IPMN - intraductal papillary mucinous neoplasm; MCN - mucinous cystic neoplasm)

Papanicolaou society of cytopathology system for reporting pancreaticobiliary cytology	
I. Non-diagnostic	
II. Negative (for malignancy)	Benign pancreatic tissue Acute, chronic or autoimmune pancreatitis Pseudocyst, lymphoepithelial cyst Splenule/accessory spleen
III. Atypical	
IV. Neoplastic - Benign	Serous cystadenoma Neuroendocrine microadenoma Lymphangioma
IV. Neoplastic - Other	Well-differentiated NET IPMN, all grades of dysplasia MCN, all grades of dysplasia Solid-pseudopapillary neoplasm
V. Suspicious (for malignancy)	
VI. Positive or Malignant	Ductal adenocarcinoma of the pancreas and its variants Cholangiocarcinoma Acinar cell carcinoma Poorly differentiated (small and large cell) NET Pancreatoblastoma Lymphoma Metastatic malignancy

### Data collection and statistics

The medical documentation of patients was collected using MedSolution medical recorder. Statistical analysis was performed using SPSS software version 22 (SPSS Inc., Chicago, Illinois, USA) and SigmaPlot 12.5 (Systat Software Inc., San Jose, California, USA). Values of *p* < 0.05 were considered significant. The differences in the bloodiness, cellularity, number of smear pairs per puncture and diagnostic smear pairs between the SS and SP group were compared using paired sample t-tests and Mann-Whitney Rank Sum Test. We used logistic regression analysis, Fisher’s exact test and Chi Squared test to identify the factors that can modify the effectiveness of sampling. Descriptive statistics were expressed as mean and median with ranges.

## Results

### Characteristics of patients and sampling

92 EUS-FNA sampling of 89 patients were involved between January 2014 and June 2016. Sampling had to be repeated in three patients due to non-diagnostic smears. There was no significant proportional variance with regard to the patients’ gender: male-female ratio was 38:51. Mean age at time of sampling was 66.1 years (range 27–95; median 69). Lesions were located most frequently in the pancreatic head (*N* = 71; 79.8%). The mean diameter was 31.8 mm (range 7–62; median 30), and in 44 cases (47.2%) cancer antigen 19–9 (CA19–9) was elevated. The characteristics of patients and EUS-FNA examinations are summarized in Table 3.

**Table 3:** Baseline characteristics of patients and sampling (CA19–9 – carcinoma antigen 19–9; CgA – chromogranin A; LMWH – low molecular weight heparin; PAI – platelet aggregation inhibitors)

Patients (*N* = 89)		Sampling (*N* = 92)	
Male/female	38/51	Examiners: Z.Sz/L.Cz	70/22
Age (year)	66.1 (27–95; median: 69)	Punctures per examination	4 (3–7; median: 4)
Tumor location		3 2 punctures 4 punctures 5 punctures 6 punctures 7 punctures	23 (25%) 47 (51.1%) 17 (18.5%) 6 (4.3%) 1 (1.1%)
Head Body Tail Diffuse	71 (79.8%) 7 (7.9%) 8 (8.9%) 3 (3.4%)		
Tumor size (mm)	31.8 (7–62; median: 30)	LMWH PAI	10 (10.9%) 14 (15.2%)
CA 19–9			
Elevated Normal No data CgA elevation	44 (47.2%) 27 (29.3%) 18 (19.5%) 5 (5.6%)	Needle type Echotip EZ Shot 2	37 (40.2%) 55 (59.8%)

There was no significant difference between the use of Cook Medical and Olympus EZ Shot 2 needles (47 vs. 55 cases). The mean number of passes for each lesion was 4 (3–7; median 4).

### Comparison of diagnostic yield of EUS-FNA sampling using SS and SP

**Fig. 1:**
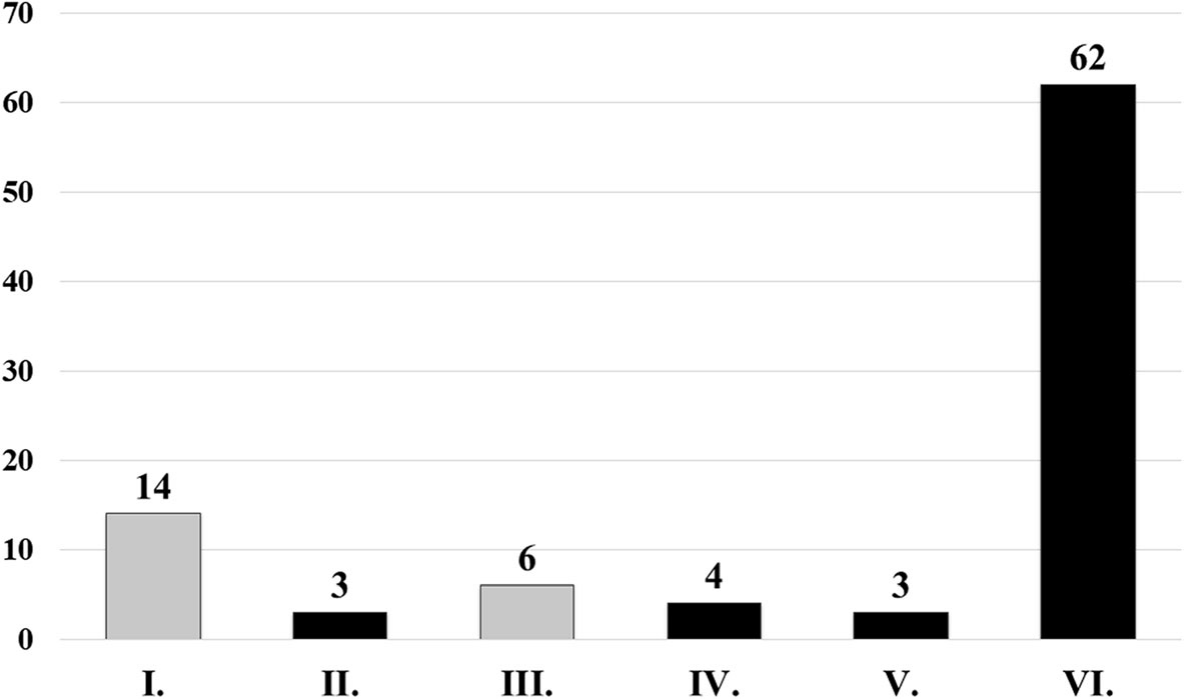
Efficacy of sampling according to the classification of Papanicolaou Society. Grey color shows the non-diagnostic and black the diagnostic categories

**Fig. 2:**
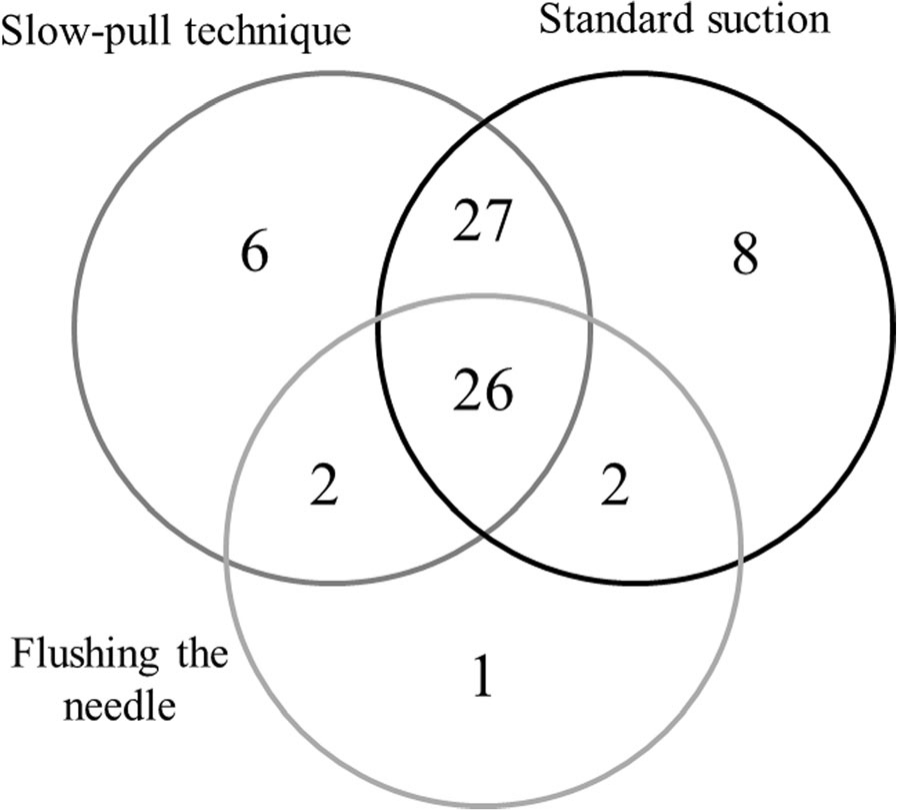
Venn diagram: distribution of diagnostic samples obtained by the flushing of the needle, SS and SP techniques

EUS-FNA sampling was diagnostic in 72 cases (78.3%): the presence of neoplasm was confirmed in 69 cases (Papanicolaou IV, V, VI) and chronic pancreatitis in 3 cases (Papanicolaou II). *(*Fig. 1*)* There was no significant difference between the diagnostic yield of SP and SS (65.2% vs. 67.4%), although the technical success rate was higher in the SS group (92.4% vs. 100%), but it was not statistically relevant. *(*Table 4*)* Cytological examination of the fluid obtained by flushing the needle with saline confirmed the diagnosis in 31 cases (33.7%), and in one patient the diagnosis was based only on this cytological sample. Figure 2 shows the distribution of diagnostic slides between the three techniques (flushing the needle, SS and SP). Histological samples were taken in 60 cases with similar efficiency in the SS and SP group (50.0% vs. 53.3%). There was no detectable difference between the two groups in the diagnostic yield of histological samples (58.7% vs. 63.2%). The diagnostic yield of EUS-FNA examination was not influenced by the endosonographer, the needle type, tumor size and location.

**Table 4:** Comparison of standard suction and stylet capillary technique

	Capillary Technique	Standard Suction
Technical success rate	85 (92.4%)	92 (100%)
Diagnostic yield	60 (65.2%)	62 (67.4%)
Histological sample obtained	49 (53.3%)	46 (50.0%)
Diagnostic yield of histological sample	31 (63.2%)	27 (58.7%)

**Fig. 3:**
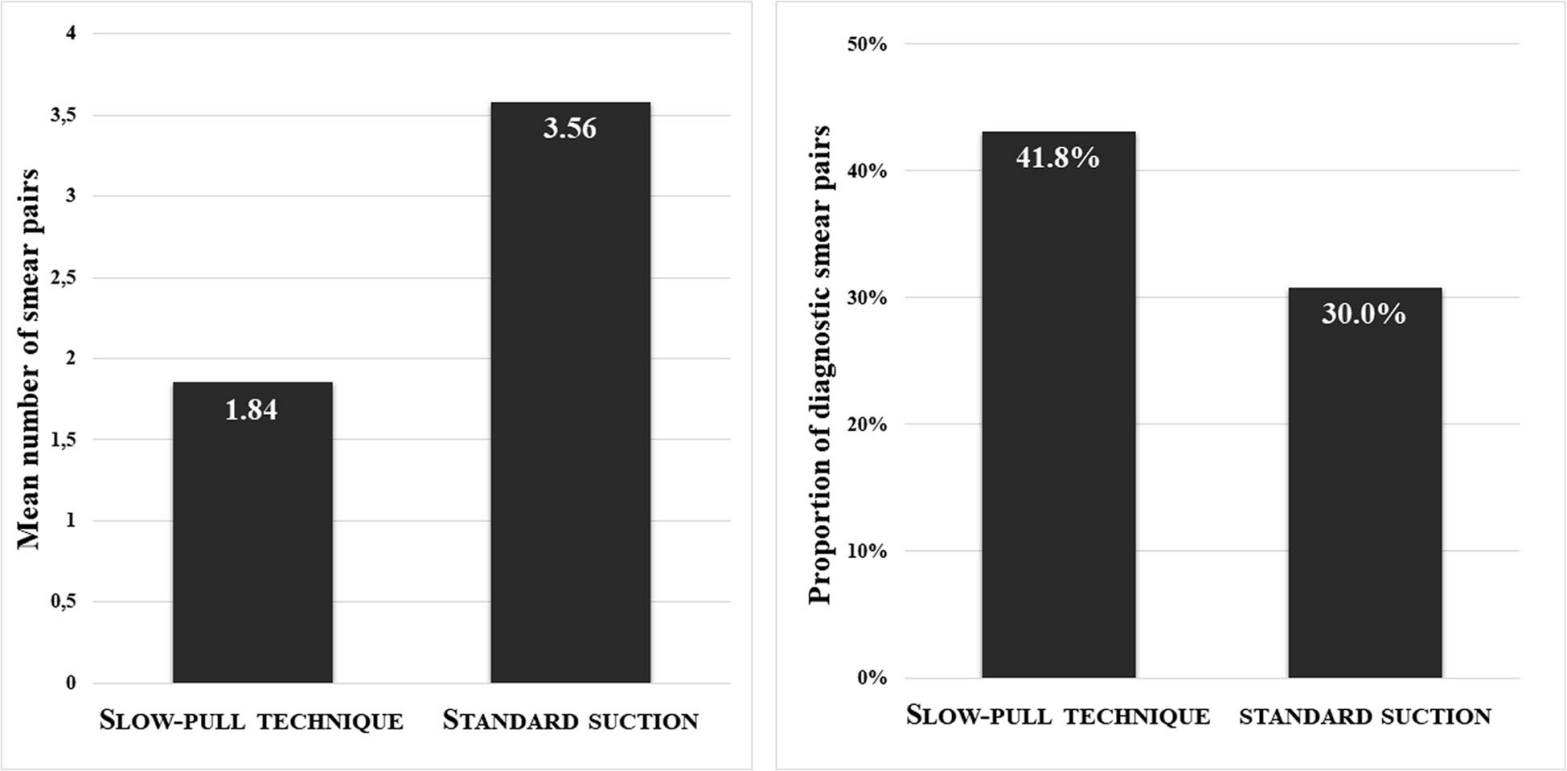
The mean number of smear pairs per puncture obtained by standard suction was significantly higher (*p* < 0.001) and the proportion of diagnostic slides was lower compared whit stylet capillary technique (*p* = 0.003)

**Fig. 4:**
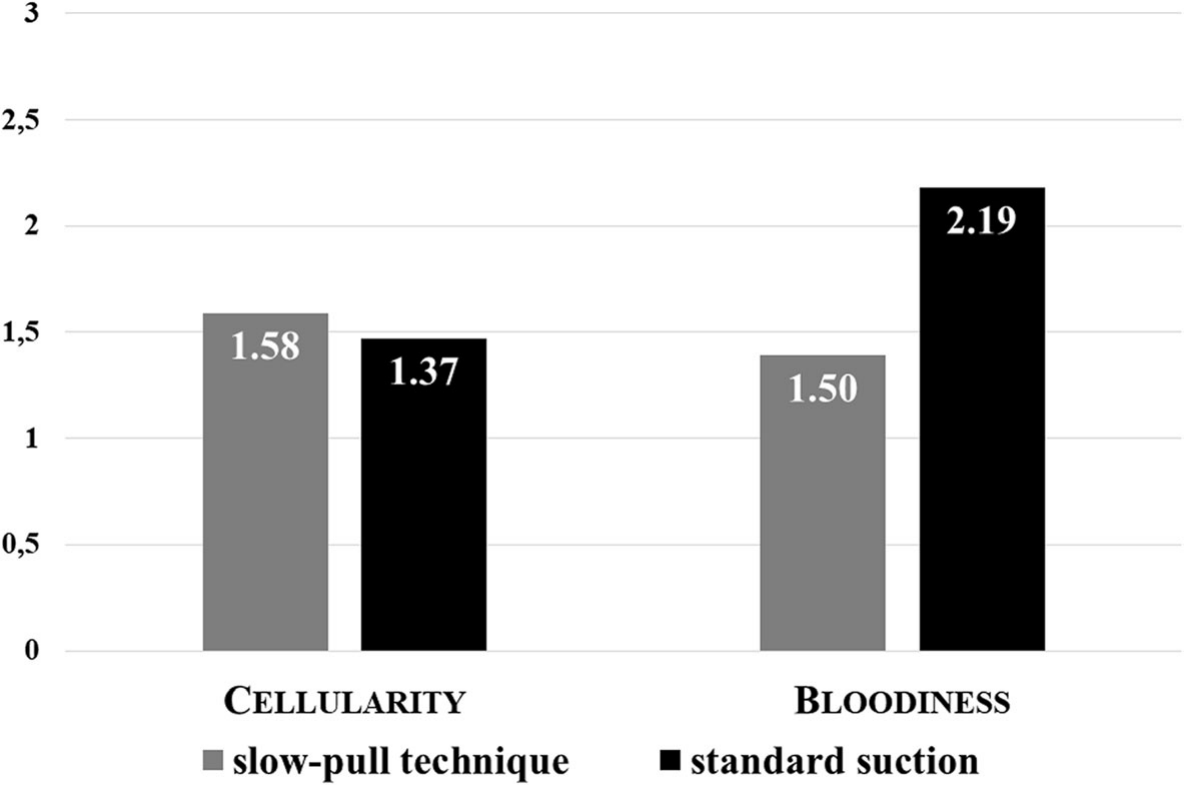
There was no difference between the cellularity of smears obtained by stylet capillary technique and standard suction, but the bloodiness was significantly higher in the standard suction group (*p* < 0.001)

The average number of smear pairs for one pass was significantly higher in the SS group (3.56; range 1–9.5; median 3.5) compared with the SP one (1.84; range 0–7.5; median 1.5), but it was associated with considerably increased bloodiness (1.50 vs. 2.19; *p < 0.001*). Cellularity did not differ statistically between the groups (1.58 vs. 1.37; *p* = 0.2554). In contrast, the proportion of diagnostic smears obtained with SP was higher (41.8% vs. 30.0%; *p = 0.003*). *(*Fig. 3-4*).*

### Complications

Early complication occurred in three cases (3.2%). A 66-year old man developed mild, postprocedural acute pancreatitis, which recovered during 4-days long total parenteral nutrition. Two patients had threefold elevation in serum amylase level without clinical symptoms. Severe and late complications were not found.

### Follow-up of patients

In 69 of 72 diagnostic EUS-FNA samplings pathological examination demonstrated neoplastic pancreatic lesions. Ductal adenocarcinoma was the most frequent neoplasm with 64.1% incidence rate. Five low grade and one high grade neuroendocrine tumors (NET) were identified (6.5%). The latter was proved to be ductal adenocarcinoma by autopsy. On one occasion signet ring cell carcinoma was found. Repeated histological sampling (5 autopsies, 8 surgical samples, 2 transabdominal core biopsies) confirmed the results of EUS-FNA in 14 cases, and in the rest 55 cases the clinical course affirmed the diagnosis. In a case of chronic pancreatitis based on the FNA results, Whipple procedure was performed due to biliary obstruction. Pathological evaluation of the surgical specimen revealed pancreatic intraepithelial neoplasia 1B (PanIN-1B) besides chronic pancreatitis.

In 5 out of 14 non-diagnostic EUS-FNAs (Papanicolaou I) benign disease was detected by further examinations (1 autoimmune and 3 chronic pancreatitis; 1 infection). In the rest 9 cases ductal adenocarcinoma (*N* = 5), biliary duct carcinoma (*N* = 1), intraductal papillary mucinous neoplasm (IPMN; *N* = 1) and metastatic renal cell carcinoma (*N* = 2) were identified. In the background of atypia benign disorder was found only in one case (autoimmune pancreatitis).

Based on the results of follow-up the sensitivity, specificity, negative predictive value (NPV), positive predictive value (PPV) and accuracy of EUS-FNA sampling in the identification of pancreatic neoplasm were 83.1, 100, 39.1, 100 and 84.8%, and no detectable difference was found between the SP and SS groups (69.9, 100, 26.5, 100 and 84.8% vs. 73.5, 100, 29, 100 and 76.1%). *(*Table 5*).*

**Table 5:** Efficacy of EUS-FNA in the identification of pancreatic malignancy (NPV – negative predictive value; PPV – positive predictive value)

Efficacy of Eus-FNA in the identification of pancreatic neoplasm			
	Independently from the method	Slow-Pull	Standard suction
Sensitivity	83.1%	69.9%	73.5%
Specificity	100%	100%	100%
NPV	39.1%	26.5%	29%
PPV	100%	100%	100%
Accuracy	84.8%	72.8%	76.1%

## Discussion

Only few studies with contradictory results have been published which assessed the diagnostic yield and the quality of smears obtained by EUS-FNA of solid pancreatic masses using SS and/or SP. The important advantage of our prospective study is the use of standardized conditions. The sampling efficacy of the two techniques was assessed in the same lesions; therefore, there was no difference in the tumor characteristics (size, location, vascularity, stiffness) and in the diagnostic process (premedication, endoscope, needle type and diameter, endosonographer, assistant, pathologist, etc.).

The diagnostic accuracy of EUS-FNA sampling was 78.3%. No difference was found between the two techniques (SS: 67.4% vs. SP: 65.2%); but higher diagnostic accuracy could be achieved with the combination of the techniques than with one technique alone. These results correlate with previous studies. The retrospective observational study carried out *Touchefeu* et al. followed 100 consecutive patients with suspected malignant pancreatic masses, and concluded that EUS-FNA provides accurate diagnosis in about 80% of cases, and its results directly influenced the management strategy in 62% of cases [13]. The meta-analysis of *Hewitt* et al. assessed the data of 4984 EUS-FNA examination from 33 papers, and found that the pooled sensitivity and specificity of malignant cytology is 85% (95% CI, 84–86) and 98% (95% CI, 97–99), respectively [4]. Previous trials also suggest that the efficacy of EUS-FNA sampling could be influenced by the sampling process and the type of suction. The results of two prospective studies showed that capillary technique with 22G needle improves the histological quality of samples, which is manifested in a higher proportion of tissue microfragments, but does not result in elevated diagnostic accuracy compared with suction technique [9, 14]. In contrast, *Puri* et al. found significantly higher sensitivity and negative predictive value when suction technique with 22G needle was applied (85.7% vs. 66.7%), but it was also associated with increased number of slides (17.8 ± 7.1 vs. 10.2 ± 5.5; *p = 0.001*) and higher blood-contamination [10]. The study performed by *Lee* et al. also confirmed these results. They assessed the results of 324 punctures from 81 patients and found significantly higher diagnostic yield (85.2% vs. 75.9%; *p* = 0.004), sensitivity (82.4% vs. 72.1%; *p = 0.005*), cellularity (*p < 0.001*) and blood-contamination (*p < 0.001*) in the suction group, with no difference in terms of specificity (96.8% vs. 100%). The substantial disadvantage of this study was the lack of distinction between the 22G and 25G needles [15]. *Nakai* et al. detected a difference between capillary and suction technique only when 25G needle was applied (90% vs. 67%) [16]. *Dabizzi* et al. also found significantly superior adequacy rate with capillary technique compared with suction (aspiration) technique using a 25G needle (80% vs. 97%; *p = 0.04*), but they provided similar results in cellularity and blood amount [17]. Irrespective of the sampling technique, previous publications suggested that the 25G needle would be more effective than the 22G one [18–20]. A meta-analysis assessing data of 1292 patients found higher sensitivity in cases when 25G needle was used compared with the 22G needle (93% vs. 85%), but the specificity was similar (97% vs. 100%) [5]. In our study we used only 22G needles, and we detected significantly higher blood contamination and lower cellularity in the SS group, but the technical success rate and the diagnostic accuracy did not show statistically relevant difference.

Immunohistochemistry is often essential for the accurate diagnosis of nonmalignant lesions and for precise subtyping of uncommon pancreatic neoplasms, such as mesenchymal, neuroendocrine, solid pseudopapillary tumors or metastases. Contrary to the conventional smears, which usually yield only limited material available for ancillary techniques, FFPE tissue blocks allow multiple sections to be cut from it allowing multiple immunostains. In our study, histological samples could be obtained in 65.2% of the cases with similar proportion in the SS and SP groups (50.0% vs. 53.3%). The distinction between tissue fragments and coagulum based on the macroscopic appearance was not possible in the majority of the cases; therefore, in most of nondiagnostic cases formalin fixed samples contained only red blood cells. The diagnostic accuracy of tissue samples was 66.7%, which is consistent with the results of previous publications [21]. In a prospective study carried out by *Kida* et al. the tissue sampling rate for histology was 83% with a diagnostic accuracy of 85%, which was not influenced by the needle size (22G 58%; 25G: 56%). Despite the significantly higher sampling rate for cytology, the diagnostic accuracy of histology and cytology did not differ significantly from each other: they were 66 and 75% with the 22G needle vs. 73 and 60% with the 25G needle [22]. *Park* et al. concluded that combined analysis is more sensitive than cytology and histology alone (81.8% vs. 69.8% vs. 67.2%; *p < 0.01*) [23]. *Hucl* et al. showed that the average number of passes to obtain sufficient tissue is significantly lower when 22G ProCore needles were used compared with the standard 22G needles (1.2 ± 0.5 vs. 2.5 ± 0.9; < 0.001) [24], but the diagnostic accuracy did not show relevant difference in the two groups [25]. *Vanbiervliet* et al. confirmed these results, and additionally found that the overall sample quality was significantly better in case of standard 22G needle [26]. Our previous experiences did not confirm the superiority of ProCore needles compared with the standard needles, therefore we used them only in case of suspicious autoimmune pancreatitis, neuroendocrine and stromal tumors.

The higher cost compared to transabdominal sampling is one of the important disadvantages of EUS-FNA. The price of the endoscopic ultrasound system and the needles in themselves are outstandingly high; the relatively high number of stained smears further increases the overall costs. The technique which reduces the number of samples without impairing the accuracy could make EUS-FNA sampling more cost-effective. Rapid on-site evaluation (ROSE) may be a good alternative. It could result up to 3.5–15% improvement in adequacy rates and accuracy of the cytological examination, and it could help to reduce the number of EUS-FNA passes and slides, which could further shorten the length of examination and pathological evaluation [13, 27–29]. *Fabbri* et al. found that tissue samples obtained by ProCore needles could achieve comparable adequacy and diagnostic accuracy with rapid on-site evaluation (ROSE) [30], and it could be more cost-effective [31]. ROSE was not available during the study period in our department, and previously we did not experience better adequacy rates with ProCore needles. However, capillary technique resulted in significantly lower number of smears without any impairment in cellularity and diagnostic yield.

The complication rate of EUS-FNA is approximately 1%, and includes infection, bleeding, and acute pancreatitis [7, 32]. In our study it was slightly higher (3.2%), but in two cases the elevation of serum amylase level was not accompanied by clinical symptoms of pancreatitis, and only one patient had mild, acute pancreatitis (1.1%). Based on these results, our study confirmed the safety of EUS-FNA sampling.

The relatively small number of enrolled cases was a major limiting factor. Not being a randomized controlled trial is another drawback of our study. The order of techniques was not randomized, SCT was performed first in all of the cases, which could possibly influence the smears’ quality – this effect might have been avoided with randomization.

## Conclusion

This study revealed that SP is an effective method with an outstanding technical success rate and efficacy compared to SS in the EUS-FNA sampling of pancreatic lesions. Cellularity of smears and the rate of acquiring sufficient histological material are similar with SS; however, lower bloodiness of samples and decreased number of slide pairs may result in faster pathological diagnosis and more cost-effectiveness in case of SP. Thus, we recommend SP as the first sampling method of solid pancreatic lesions. It may further be supplemented by SS in case no tissue fragments could be obtained or when the macroscopic appearance of the samples suggests their inappropriateness for the diagnosis based on the consideration of the endosonographer.
